# Surface Interactions and Nanoconfinement of Methane and Methane plus CO_2_ Revealed by High-Pressure Magic Angle Spinning NMR Spectroscopy and Molecular Dynamics

**DOI:** 10.3390/membranes12121273

**Published:** 2022-12-15

**Authors:** Salim Ok, Siddharth Gautam, Kao-Hsiang Liu, David R. Cole

**Affiliations:** 1 School of Earth Sciences, The Ohio State University, Columbus, OH 43210, USA; 2 Shull Wollan Center—A Joint Institute for Neutron Science Oak Ridge National Laboratory, Oak Ridge, TN 37831, USA

**Keywords:** natural gas, confinement, nanoporous silica, methane, high-pressure NMR, MD simulations

## Abstract

This study explores the fundamental, molecular- to microscopic-level behavior of methane gas confined into nanoporous silica proxies with different pore diameters and surface-to-volume (S/V) ratios. Surfaces and pore walls of nanoporous silica matrices are decorated with hydroxyl (-OH) groups, resembling natural heterogeneity. High-pressure MAS NMR was utilized to characterize the interactions between methane and the engineered nanoporous silica proxies under various temperature and pressure regimes. There was a change in the chemical shift position of confined methane slightly in the mixtures with nanoporous silica up to 393 K, as shown by high-pressure ^13^C-NMR. The ^13^C-NMR chemical shift of methane was changed by pressure, explained by the densification of methane inside the nanoporous silica materials. The influence of pore diameter and S/V of the nanoporous silica materials on the behaviors and dynamics of methane were studied. The presence of CO_2_ in mixtures of silica and methane needs analysis with caution because CO_2_ in a supercritical state and gaseous CO_2_ change the original structure of nanoporous silica and change surface area and pore volume. According to simulation, the picosecond scale dynamics of methane confined in larger pores of amorphous silica is faster. In the 4 nm pore, the diffusivity obtained from MD simulations in the pore with a higher S/V ratio is slower due to the trapping of methane molecules in adsorbed layers close to the corrugated pore surface. In contrast, relaxation measured with NMR for smaller pores (higher S/V) exhibits larger T_1_, indicating slower relaxation.

## 1. Introduction

The properties of bulk fluids are altered by solid substrates, confinement between two solid surfaces, or in narrow pores because of the interplay of the intrinsic length scales of the fluid and the length scale due to confinement and potential interactions between surfaces such as pore walls and the fluids [1]. The behavior of fluids (i.e., gases and liquids) in confined geometries (pores, fractures) exhibit several deviations from their bulk behavior. Thermodynamics such as phase transitions (i.e., freezing and capillary condensation), sorption and wetting, and dynamical properties, including diffusion and relaxation, can be altered, with the most substantial changes observed for pores ranging in size from <2 nm to 50 nm—the micro- and mesoporous regimes. The factors affecting the structure and dynamics of the confined fluids include surface-to-volume ratios, the average pore size distribution, the level of pore interconnection, and the strength of the fluid-substrate interaction. A quantitative understanding of the complex solid-fluid interactions under different thermodynamic conditions will influence both the design of better substrates for technological applications (e.g., chromatography, fluid capture, storage and release, and heterogeneous catalysis) as well as our fundamental understanding of the engineered processes (i.e., fluid and waste mitigation, carbon sequestration, gas shale recovery, etc.).

Determining the origin, migration, and trapping behavior of volatile hydrocarbons in the geo-energy subsurface systems is of economic interest. Indeed, industry exploration and exploitation of shale gas (e.g., the Marcellus, Utica, and Barnett formations) have focused on understanding the fundamental behavior of volatile hydrocarbon—rock matrix interactions [2]. Hydrocarbon fluids, including methane (most abundant), ethane, and other longer chained alkanes, are stored in three forms: free gas in pores, free gas in natural fractures, and adsorbed on organic matter and silicate mineral surfaces [3]. The pores are typically submicron in size, cylindrical or slit-like shape, and commonly dominated by those as small as a few nm [4]. Understanding the molecular characteristics of methane structure and dynamics in narrow silica-based pores is necessary for quantifying the molecular phenomena relevant to natural gas production following hydraulic fracturing. Several subsurface phenomena, including hydrocarbon migration, can be better understood and predicted once the adsorption and diffusion of hydrocarbons in narrow pores are constrained [5]. Understanding methane behavior in little pores can also impact industrial processes such as utilizing catalytic materials. 

Several experimental and computational techniques have been used to study fluids under confinement. In particular, the synergistic use of neutron scattering, NMR experiments, and molecular dynamics simulations has added immensely to understanding fluid behavior under confinement [6]. The behavior of confined methane has been studied using a combination of NMR and MD simulations [7]. MD simulations have also been used to study the structure–dynamics correlations in methane confined in silica pores [8].

### Background and Objectives

Methane gas behavior has been investigated in silicoaluminaphosphate (SAPO) [9] and in aluminophosphates (AlPO) [10] molecular sieves possessing sizeable internal surface area to adsorb guest molecules of proper size and shape. Methane/propane hydrates formation was investigated by ^13^C NMR [11]. However, in the studies above, high pressures above and below the critical pressure of methane were not studied in detail due to the lack of analytical instrumentation that can provide and handle high-pressure operational conditions. In other words, these studies did not preserve in situ P-T conditions during methane-hydrate-bearing sediment recovery but—like geophysical techniques and geotechnical testing of synthetic sediments—relied instead on measuring mechanical and hydraulic properties (like stiffness, shear strength, and dilatancy) with increasing hydrate saturation [12].

A recently established high-pressure magic angle spinning (MAS) high-pressure NMR method allows for studying methane behavior in confined states at pressures such as 60 and 120 bar and temperatures as high as 393 K [13,14,15]. Initially, MAS NMR was developed to investigate substances, materials, such as polymers, and molecules in the solid state of matter when the samples are not soluble in common organic solvents. The primary goal of MAS NMR was to remove “the orientational dependence of the NMR anisotropic interactions” of the samples in the solid state [16]. In MAS, if the sample is fast enough, i.e., 5–65 kHz [17], with an angle of 54.74° around an axis by referencing the static magnetic field, the result is averaging the anisotropy of nuclear interactions that show frequency dependence on the orientation according to a second order Legendre polynomial. Advancements in solid-state NMR made MAS a daily routine method for studying solid samples with high-resolution NMR spectra. However, till the 2000s, investigating behaviors of gases by MAS NMR was scarce [18]. In 2006, Deuchande et al. designed and showed the performance of a high-pressure insert made up of the polymer (poly ether ether ketone) (PEEK) [19]. In the last decade, Hoyt et al. [13] and Turcu et al. [14] improved the approach of MAS NMR, into which gas was loaded with a chamber allowing pressurization of the gas samples, including methane and carbon dioxide (CO_2_). Recently, high-pressure MAS NMR has been applied to study methane and CO_2_ in mixtures with natural clays, such as smectites [20,21], natural shale [22,23], as well as clay swelling in dry supercritical CO_2_ [24].

Recently, the new high-pressure MAS NMR technique has been applied in several studies on light hydrocarbon-subsurface energy systems of both engineered proxies and minerals. Bowers et al. focused on the interaction of methane with the natural San Bernardo hectorite (SHCa-1) with different wetting degrees by using various high-pressure techniques, including the high-pressure MAS-NMR method [20]. The results suggested that under subsurface conditions of approximately 1 km depth at 90 bar and 323 K, smectite interlayers might function as a host for methane if there is a low volume of water. As shown by Walter et al. [25], it was possible to monitor the hydrogenolysis of benzyl phenyl ether, catalyzed with Ni/γ-Al_2_O_3_ by high-pressure MAS-NMR. High temperature/pressure MAS-NMR has been utilized by Chamas et al. [26] to study the adsorption and confinement of supercritical CH_4_/CO_2_ in clays and other minerals, revealing pressure-dependent ^13^C chemical shifts.

Further, Bowers et al. [27] demonstrated three environments for CH_4_ upon mixing CH_4_ with smectite clays: bulk fluid, in the interparticle pore spaces, and the interlayer nanopores of the clays. The ^13^C NMR chemical shift position became more positive when the accessible physical area of the clay for CH_4_ was decreased. Bowers et al. [28] focused on probing the dimension and connectivity of engineered nanoporous silica with pore sizes of 2.5, 5.0, 10.0, and 20.0 nm by applying high-pressure ^13^C MAS-NMR and using supercritical CH_4_. When the pore diameter was increased, the ^13^C shift of supercritical CH_4_ adsorbed in the nanoporous silica materials became more negative. Exposure of H_2_O to the system resulted in the occupation of smaller pores by H_2_O molecules; hence, CH_4_ fills the larger pores. There was the exchange of CH_4_ molecules between nanopores and bulk fluid environments. However, the presence of H_2_O decreased the distribution of exchange rates between CH_4_ molecules in the pores and bulk. In the contribution of Bowers et al. [27], a natural clay was utilized for confining methane at a constant pressure of 90 bar. In contrast, we confined methane into synthetic engineered proxies of nanoporous silica and varied the pressure. Compared to Bowers et al. [28], we utilized white powder nanoporous silica down to a pore diameter of 1.5 nm with relatively higher surface areas than porous monolith disks. In the present contribution, we tested the influence of CO_2_ in gaseous and supercritical states along with computational efforts, which are not explored in the reports by Bowers et al. [27,28].

The objective of the present study is to provide fundamental, molecular- to microscopic-level descriptions of the methane within nanoporous silica with 1.5 nm and 2.5 nm of pore diameter exhibiting differences in surface-to-volume ratios, surface area, and ordered structure [29]. The materials from the same batch as in Ref. [29] are used, confirming that the materials have comparable pore size and surface area/volume characteristics as reported previously. The preparations of these materials are described in detail elsewhere [30,31]. Both nanoporous silica samples used in the present study resemble natural environments because of heterogeneity arising from the decoration of their surfaces and pore walls with hydroxyl groups [15,32,33]. In the current contribution, the two nanoporous silica materials were used as subsurface model systems for exploring nanoconfinement behaviors and dynamics of carbon-13 labeled methane gas. To study methane behavior under nanoconfinement, we used a recently developed high-pressure MAS NMR system [13]. Molecular simulations have been conducted to explain the experimental results. Hence, we aim to achieve the following specific goals: (i) to determine the degree of deviation of confined methane behavior compared to bulk methane, (ii) to clarify the influence of pore parameter and surface-to-volume ratio on the deviation of confined fluid behavior from bulk by also referring to a previous publication where methane was mixed with nanoporous silica with 4.0 nm of pore diameter [15], (iii) to explore the effect of CO_2_ on methane behavior and nanoporous silica, and (iv) to show how to differentiate signals of confined fluid in nanopores from the excess interparticle fluid.

## 2. Materials and Methods

### 2.1. Materials

The two mesoporous materials, 2.5 nm silica and 1.5 nm silica, have surface-to-volume ratios of 1203 and 693 cm^−1^, respectively. These materials were characterized by different techniques, including Transmission X-ray diffraction (XRD), thermogravimetric analysis (TGA), and Brunauer–Emmett–Teller (BET) surface area analyzer, as detailed previously [29].

### 2.2. Nuclear Magnetic Resonance 

The experimental setup of the high-pressure MAS NMR has been described in detail [13,14,15]. The probe volume of the 7.5 mm outer diameter rotor is 446 μL [14]. High-pressure 7.5 mm NMR rotors were spun at a 3 kHz rotating frequency, and ^1^H-decoupled ^13^C NMR spectra were acquired using direct polarization experiments by averaging 120-time domain transients. The data were obtained on a Varian NMR system with a 7.0 T magnet using a 7.5 mm HX probe. Temperature calibration of the high-pressure system was accomplished by acquiring ^207^Pb NMR spectra of lead nitrate as a function of the spectrometer temperature setting [34,35]. Note that the two different loading pressures are 30 and 120 bar, below and above supercritical pressure conditions, and the experiments are conducted at two temperatures of 35 and 75 °C. The samples for ^13^C NMR spectra were prepared at loading pressures of approximately 30.0, 60.0, and 120.0 bar and 323 K ± 1.0 K. Two temperatures were employed for studies at each pressure (307 and 346 K ± 1.0 K) for ^13^C NMR spectra yielding internal sample pressures of 28.2, 56.4, 112.7, or 32.6 bar, 65.1 bar, 130.3 bar (±0.3 bar), respectively, as calculated previously [15]. Varying the pressure produced a change in the density of methane and provided the opportunity to interrogate the impact that phase change (gas to supercritical state) had on the fluid-silica interaction. The critical pressure (P_c_) and critical temperature (T_c_) of methane are 45.992 bar and 190.564 K (−82.7 °C), respectively [15]. Thus, the temperature–pressure (density) regimes accessible with the high-pressure MAS technique are consistent with those identified for shale gas systems encountered at the specified depths in the earth’s subsurface. The standard Varian saturation recovery pulse sequence was utilized to measure the longitudinal magnetization relaxation times (T_1_).

### 2.3. Molecular Simulations

To provide a molecular underpinning to the physical and chemical behavior of the methane species studied with NMR experiments, molecular dynamics simulations were carried out considering 1.5, 2.5, and 4.0 nm cylindrical silica pore models constructed using an approach similar to earlier studies [36,37,38,39]. This approach involves melting a crystalline SiO_2_ simulation box by heating it to high temperatures and cooling it to obtain an amorphous silica box. This is followed by drilling a cylindrical pore by removing atoms within a cylindrical region. Initially, an α-cristobalite unit cell [40] was replicated 12 × 12 × 10 times to get a simulation cell of crystalline SiO_2_. This simulation cell was heated to 5000 K by carrying out an NPT simulation at 1 bar lasting 100 ps with one fs time step. ClayFF force field [41] was used to model the interaction between the Si and O atoms in this simulation. After the simulation cell was melted, it was cooled to 300 K by running subsequent NPT simulations, each lasting 100 ps at 4000, 3000, 2000, 1000, and 300 K. The input in all these simulations, was obtained from the output of the previous simulations. This cooling in steps helps avoid the build-up of unwanted stress. Finally, an NVT simulation of 100 ps at 300 K was carried out with the simulation cell obtained from the last of the simulations mentioned above. A simulation cell of amorphous silica was thus obtained. A cylindrical pore of diameter 4 nm along the Cartesian *Z*-axis was etched out from this amorphous silica by removing all atoms within a radius of 2 nm from the *Z*-axis passing through the center of the simulation cell. The same procedure was followed to obtain smaller pores, starting with a smaller crystallite simulation cell obtained by replicating a unit cell of α-cristobalite 10 × 10 × 10 times. The box length in the Z-direction (i.e., parallel to the pore axis) for all cases was 7.1 nm. For smaller pores (1.5 nm and 2.5 nm), the X and Y dimensions of the box were 5.1 nm, while that for the larger pores was 6.1 nm.

To probe the effects of surface to volume ratio of the pore, a pore of 4 nm diameter was also etched out from the larger amorphous silica cell by undulating the circular profile of the pore surface with a sinusoidal varying function. This resulted in an undulated cylindrical pore of 4 nm diameter with a higher surface-to-volume ratio than the smooth pore of the same volume described above. Undulating the circular profile of the pore surface this way gets progressively more difficult as the pore radius decreases. Therefore, we chose to study the effects of the surface-to-volume ratio in the largest of the three pore sizes (i.e., 4, 2.5, and 1.5 nm). Finally, after etching out the pores, the pore surface in each case was decorated with -OH groups by attaching -OH groups to unsaturated Si atoms and H atoms to unsaturated O atoms from SiO_2_ such that all resulting -OH pointed towards the pore axis. 

Obtaining the amorphous silica pores, methane molecules modeled in the TraPPE-UA [42] formalism as single spherical entities were loaded in the pores using grand canonical Monte Carlo (GCMC) simulations with DL-Monte [43]. DL-Monte allows the direct use of partial pressure of the adsorbed gas as a control parameter instead of chemical potential. This is made possible by calculating the selection procedures of insertion and deletion of gas molecules directly in terms of the partial pressure of the gas [44]. The starting configuration consisted of the simulation cell with the amorphous silica and methane molecules placed at the center of the pore. GCMC simulations were then carried out. During the simulation, the translation, rotation, and insertion/deletion of methane were undertaken with a probability of 0.25, 0.25, and 0.5, respectively. The interaction parameters for the methane silica pairs were calculated using the Lorentz-Berthelot mixing rules along [45] with ClayFF and TraPPE-UA force fields. A potential cut-off of 1.4 nm, as suggested for the TraPPE-UA force field, was used. Each simulation consisted of 2 million steps, the first 500,000 of which were discarded to account for initial equilibration requirements. The number of methane molecules in the simulation cell were averaged over the entire production run of 1.5 million steps corresponding to the partial pressures in the simulation. In the experiments, methane was loaded at a temperature of 323 K with various pressures specified. Therefore, to make fair comparisons with the experiments, the GCMC simulations for loading methane were also carried out at 323 K. The number of methane molecules in the pores thus obtained was used for MD simulations at 307 and 346 K without any change.

**Figure 1:**
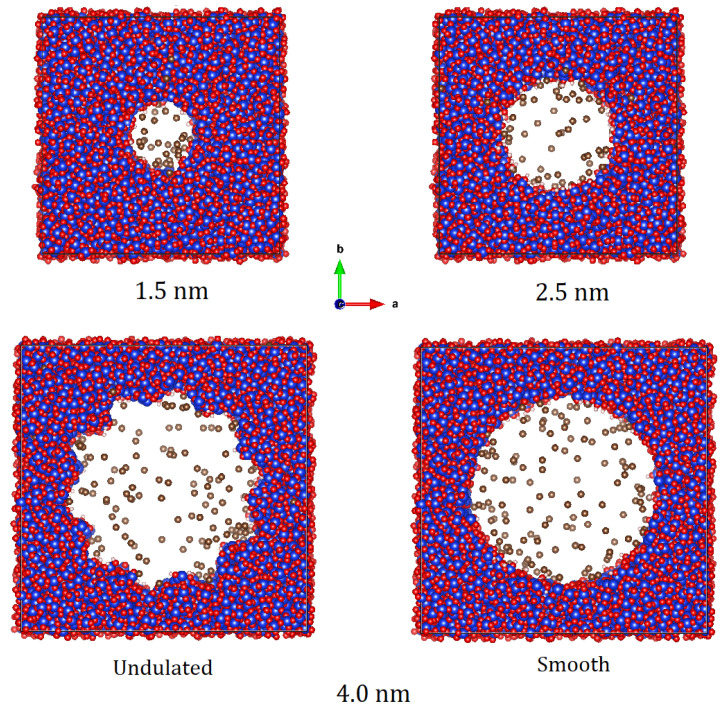
Simulation snapshots from the MD simulations of methane loaded in different pores at 323 K and 30 bar. The top two panels show the smaller pores of size 1.5 nm and 2.5 nm, while the bottom panels show the 4 nm pore with undulated and smooth pore profiles as indicated. Blue, red, white, and brown spheres represent silicon, oxygen, hydrogen atoms, and methane molecules in the united atom formalism.

United atom MD simulations on methane loaded in different pores were carried out using DL_Poly_4.06 [46] in the NVT ensemble employing a Nose-Hoover thermostat. The simulations used the same force-field parameters as in the GCMC simulations. Each simulation lasted 2 ns, the first 0.5 ns of which accounted for equilibration. Achievement of equilibration within the first 500 ps was confirmed by investigating the fluctuations in energy and temperature, which were within reasonable limits. A time step of 1 fs was used. MD simulations with methane loading corresponding to 30 bar at 323 K were carried out for the smaller pores of 1.5 nm and 2.5 nm diameter, each at two temperatures of 307 K and 346 K. As the effect of temperature on the properties of the confined methane was found to be minimal, all simulations for the 4 nm pores—both smooth and undulated—were carried out at only one temperature of 346 K. To probe the effect of pressure, simulations in the smooth 4 nm pore with methane loadings corresponding to 30 bar and 60 bar at 323 K were carried out. The impact of the surface-to-volume ratio was investigated by carrying out an additional simulation at 346 K in the undulated pore with a methane loading corresponding to 30 bar at 346 K. Simulation snapshots at the end of MD simulations carried out at 346 K on methane loaded in different pores at 323 K, and 30 bar are shown in Figure 1. A summary of all the MD simulations carried out is provided in Table 1.

## 3. Results

### 3.1. Methane Behavior 

**Figure 2:**
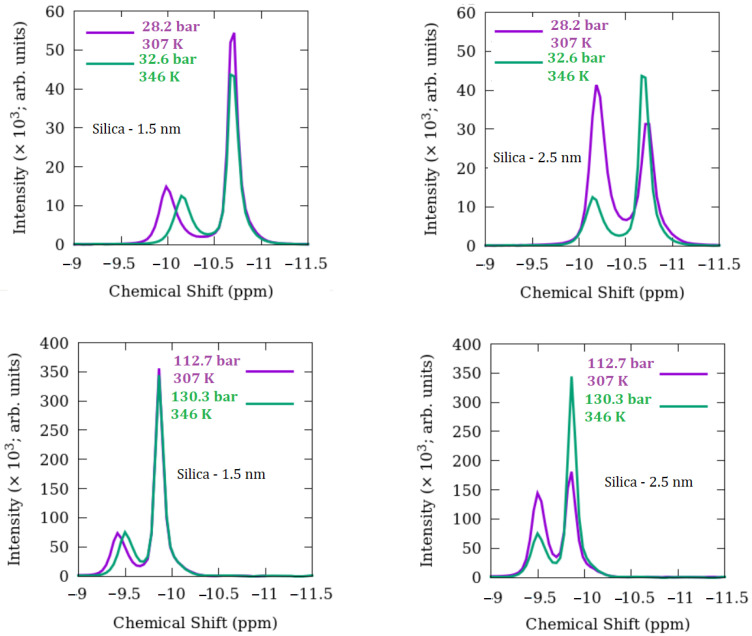
Proton decoupled ^13^C MAS NMR spectra of methane in engineered nanoporous silica.

A set of ^13^C MAS NMR experiments on bulk methane was conducted at the outset, allowing for a direct comparison of the results of methane exposed to powdered 2.5 nm silica and 1.5 nm silica. Figure 2 shows typical ^13^C-NMR spectra of methane in nanoporous silica with the two-pore diameters as a function of temperature and pressure. It is readily apparent that there are two types of peaks, one for bulk-like methane (more negative ^13^C shifts) and a second for what we classify as confined methane (less negative ^13^C shifts). Note that the P-T conditions traverse from subcritical to supercritical. Any comparisons across the P-T landscape need to account for this.

Some of the intensities and shifts could be due to phase behavior differences in methane. This is probably the case for pure bulk methane. Besides, compared to pure methane, whose spectra were acquired under the same experimental conditions, there is a line broadening in the peaks of methane molecules. The interactions of methane gas with the oxygen and hydrogen atoms in the hydroxyl groups will likely contribute to symmetry reduction [9]. Methane molecules in the mixtures with the nanoporous silica matrixes have different local environments compared to pure bulk, resulting in heterogeneity. Due to pressure, there could be densification, and some methane molecules might become liquified and interact more strongly with hydroxyl groups on the surface of the nanoporous silica materials. This might also influence the line width and cause broadening. Previously, grand canonical Monte Carlo (GCMC) simulations demonstrated that methane loading into a 4 nm silica model resulted in densities higher than methane in bulk under the same temperature and pressure conditions. Although this density changes depending on different regions of the pore, the density was the lowest close to the center of the pore and increased remarkably, getting closer to the pore walls [43].

In addition to line broadening, in mixtures of methane and 1.5 nm silica, the intensity of the peaks does not change at high pressures, and there is a slight change at lower pressures. However, in mixtures of methane and 2.5 nm silica, the intensity of the peaks changes at both high and low pressures. Important to note that at lower pressures, this change is more pronounced, and the peak assigned to confined methane has a higher magnitude than that of bulk-like methane. This is mainly attributed to the S/V ratio of 2.5 nm silica which is larger than that of 1.5 nm silica. The larger surface area of 2.5 nm silica allows more methane molecules to condense and interact strongly with the -OH on the pore walls when pressure was increased from 28.2 bar to 32.6 bar or from 112.7 bar to 130.3 bar. Hence, there is a decrease in the magnitude of the peak assigned to bulk-like methane and an increase in the intensity of the peak belonging to confined methane. 

Table 2 summarizes the measured methane ^13^C chemical shift values in the pure state and mixtures with silica systems as a function of temperature, pressure, and type of silica system (see SI of Ref. [15] for discussion of chemical shift referencing).

Figure 2 reveals some apparent trends in peak intensity for both silica materials. In comparing the results shown in Figure 2, we should consider silica materials’ surface area and pore volumes. Given that the amount of 2.5 nm silica is higher than that of 1.5 nm silica (see Table 2), 2.5 nm silica has higher pore volume and surface area than 1.5 nm silica. This is reflected in the intensities of the ^13^C NMR peaks of bulk-like versus confined methane molecules.

- Generally, bulk-like methane intensities are greater than confined peaks and greater with increased pressure except for 28.2 bar for 2.5 nm silica.
- Comparing the two silica materials, the confined methane intensities are greater for 2.5 nm versus 1.5 nm silica, and this difference is more pronounced at the lowest pressure of 28.2 bar.
- For the 2.5 nm silica, the bulk-like intensities become progressively more subordinate at the lower pressure conditions, whereas the confined intensities tend to be greater. An opposite trend is observed for the bulk-like methane in 1.5 nm silica.

The reported chemical shift of gaseous methane in the mixture with propane is −8.52 ppm at 298 K and 13.8 bar [11,15]. In our previous study [15], the isotropic chemical shift of methane in the pure state ranged between −10.45 ppm and −9.71 ppm from 28.2 bar to 130.3 bar of pressure, respectively. As with the peak intensities, we observe some systematic trends from a comparison in ^13^C chemical shifts between pure methane and bulk-like methane in the silicas and for a given silica material and between the two materials.

- The ^13^C shifts for pure bulk methane become less negative with increasing temperature and pressure,
- For most temperature-pressure conditions, bulk-like ^13^C shifts become more negative for both nanoporous silicas compared to pure methane except at 373 K, >130.3 bar,
- The ^13^C shifts for the 1.5 and 2.5 nm silica are nearly the same for the common pressure conditions measured,
- For both 1.5 and 2.5 nm silica, ^13^C shifts for confined methane are always less negative compared to bulk-like methane,
- For a given temperature, increasing pressure leads to progressively smaller differences between bulk-like and confined methane ^13^C shifts,
- In general, the differences in ^13^C shifts between bulk-like and confined methane decrease with increasing temperature and pressure for each silica system,
- Comparing both silica materials, there is a tendency for the difference in ^13^C shifts between bulk-like and confined methane to be slightly smaller for the 2.5 nm silica versus 1.5 nm silica.

We suggest that the bulk-like methane represents (a) methane filling the interparticle spaces with minimal sorption to the external surfaces of the silica particles and/or (b) perhaps those methane molecules located in the center of each pore shielded by more tightly adsorbed CH_4_ molecules on the pore walls.

**Figure 3:**
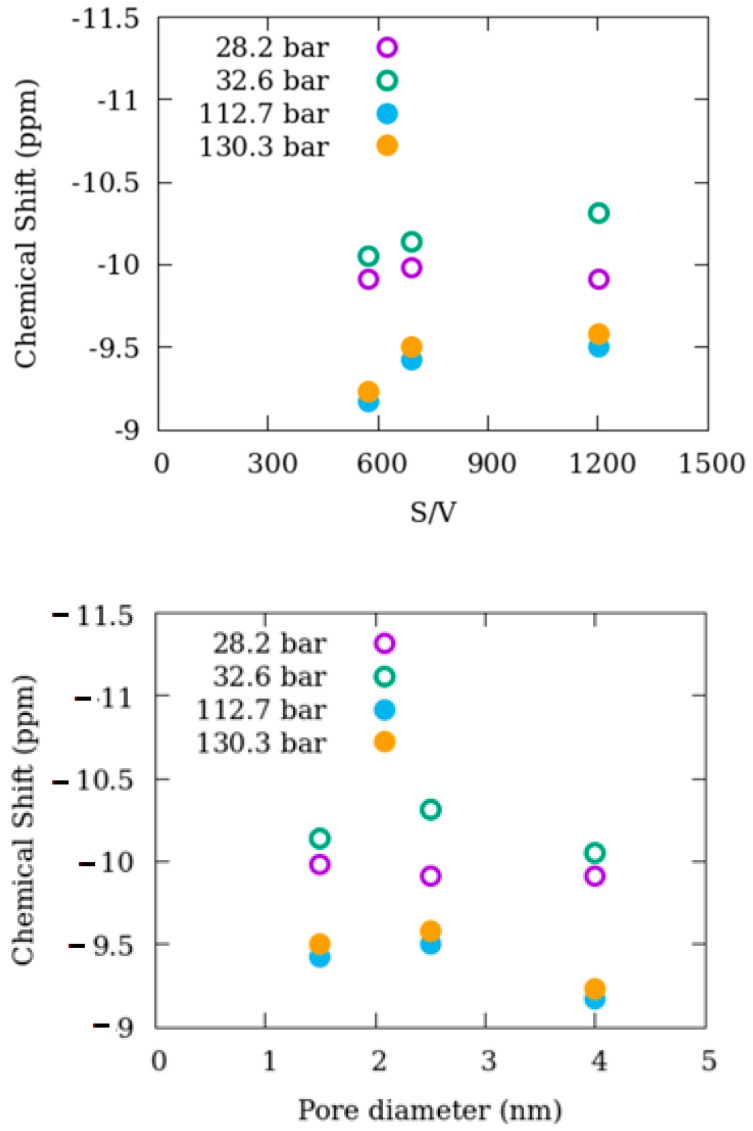
Change in the isotropic chemical shift in ^13^C NMR positions of methane in mixtures with engineered nanoporous silica proxies. The open circles represent bulk-like methane, while the filled circles represent confined methane. 307 K for P = 28.2 and 112.7; 346 K for P = 32.6 and 130.3 bar. Data on 4 nm silica-methane are from Ok et al. [15]. The unit of S/V is 1/m.

Normalization via the surface area to volume ratio (S/V) can help improve our understanding of how nanoporous silica materials affect the behavior of confined methane relative to that of bulk-like methane. Figure 3 demonstrates the changes in the isotropic chemical shift in ^13^C NMR positions of methane in mixtures with engineered nanoporous silica proxies as a function of pore diameter and S/V.

The gap in isotropic chemical shift positions of bulk-like methane and confined methane as a function of S/V is less pronounced when the S/V increases. This shows that methane molecules prefer freely moving rather than locating themselves on the surface of the pore walls by interacting with -OH groups when S/V is high, as in 2.5 nm silica. In addition, this gap is more pronounced for the 4.0 nm silica with the lowest S/V ratio [29] when the gap is compared as a function of pore diameter. The evidence indicates that the influence of 2.5 nm silica on the behavior of confined methane is smaller than that revealed by 1.5 nm silica and 4.0 silica.

### 3.2. Methane-CO_2_ Behavior

We also explored the influence of isotopically enriched CO_2_ on the behavior of methane (see Table 3) in the 2.5 nm silica. First, methane was loaded, followed by the loading of CO_2_. At a loading pressure of methane at 15 bar plus CO_2_ at 15 bar to achieve a final loading pressure of 30 bar, there was only a slight change in the isotropic chemical position of CO_2_ when the temperature was increased to 346 K from 307 K. This outcome is seen at the other loading pressures of 15 + 45 bar and 60 + 60 bar. CO_2_ does not influence the isotropic chemical shift position of bulk-like methane, as shown in Table 3. There is also no significant difference in the chemical shift position of bulk-like methane in the presence of CO_2_ and silica-2.5 nm compared to that of bulk-like methane in mixtures with silica-2.5 nm only (see Table 2). At 120 bar loading pressure, there is only a slight change in the isotropic chemical shift of confined methane in the presence of CO_2_ compared to methane only in silica-2.5 nm. At 120 bar of pressure, CO_2_ is in a supercritical state.

Conversely, in the gas state of CO_2_ at 60 bar of loading pressure, the change in the chemical shift position of bulk-like methane is 0.12 and 0.14 ppm at 307 and 346 K, respectively, compared to that of pure methane in bulk (−9.71 ppm) with a loading pressure of 120 bar [15]. Note that the amount of CO_2_ is significantly higher in the loading pressure of 60 bar than that of methane. The most significant change in the chemical shift position of bulk-like methane (0.28 ppm) is observed at 45 bar of loading pressure of CO_2_ and 307 K compared to that of bulk methane (−10.20 ppm) with a loading pressure of 60 bar [15]. This shows that in the presence of CO_2_ at higher pressures, bulk-like methane changes its behavior towards bulk methane. This suggests that when CO_2_ dominates the medium in terms of volume, confined methane molecules “compete” with CO_2_ molecules that occupy and sorb on the pore walls in the nanoporous silica material. Similar behavior was observed for propane blends with CO_2_ in silica aerogel [47]. The propane-CO_2_ QENS study in silica aerogel indicated that the presence of CO_2_ resulted in a faster diffusive behavior of propane. The interpretation is that the CO_2_ sorb to the silica aerogel surface better frees up propane in the center of the pores. An MD study of CO_2_ and CH_4_ adsorption on mesoporous silica [48] exhibited stronger binding of CO_2_ than CH_4_. This supports the assumption that CO_2_ displaces CH_4_ in mixed systems.

### 3.3. Dynamics of Methane as Revealed by NMR T_1_ Relaxation

The time needed to align the nuclei along the magnetic field direction (named the longitudinal direction) is described by a longitudinal relaxation time called T_1_ [49]. T_1_ times provide information on more localized motions of confined molecules, such as translation and rotation on a time scale comparable to the reciprocal of the NMR angular frequency (approximately 1 ns) [50]. T_1_ is governed by energy and is a measure of the dipolar interactions of the spins with the local environments [51]. 

Table 4 summarizes the T_1_ relaxation times of methane in mixtures with silica-2.5 nm and silica-1.5 nm. The T_1_ relaxation times are acquired at 308 and 348 K. In the case of silica-2.5 nm and methane, CO_2_ is also added to the mixture to explore the potential influence of CO_2_ molecules on the dynamics of methane molecules.

In the mixture of 2.5 nm silica and methane, both bulk-like and confined methane show shorter T_1_ values than pure bulk methane. However, in the mixtures with 1.5 nm silica and methane, the bulk-like and confined methane molecules demonstrated longer T_1_ values than pure bulk methane for the same conditions. This indicates that the surface-to-volume ratio, not pore diameter, has more influence on methane dynamics. Further, the presence of CO_2_ strongly affects the dynamics of methane molecules. Complicating this is that CO_2_ alters the pores in the nanoporous silica materials, leading to a change in the pore volume. There are two aspects of this result: (i) how much the nanoporous structures of silica materials change upon exposure to CO_2_, and (ii) whether CO_2_ could be utilized for natural gas or shale gas production to ease the fluid flow.

To explore the first aspect, we conducted BET surface area measurements with nanoporous silica materials after exposing them to CO_2_. The samples were outgassed at 110 °C under a vacuum before exposure to CO_2_ to eliminate water. As seen in Table 5, after exposure to CO_2_ or supercritical (sc) CO_2_ and releasing CO_2_, both surface area and pore volume of 2.5 nm silica are reduced. We suggest that during the exposure, the nanoporous silica, which is a cross-linked material, starts swelling and enlarging the pore volume leading to an increase in dynamics as revealed by longer T_1_ values.

### 3.4. Molecular Simulations 

#### 3.4.1. Structure

**Figure 4:**
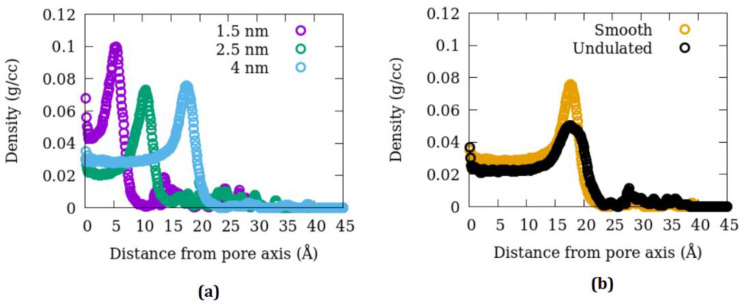
Distribution of methane in the amorphous silica pores along the radial direction moving away from the pore axis as a function of (**a**) pore size and (**b**) surface-to-volume ratio of the pore. The pore center (axis) is located at the zero of the *X*-axis. All data shown is obtained from MD simulations carried out at 346 K with a methane loading corresponding to 30 bar at 323 K.

**Figure 5:**
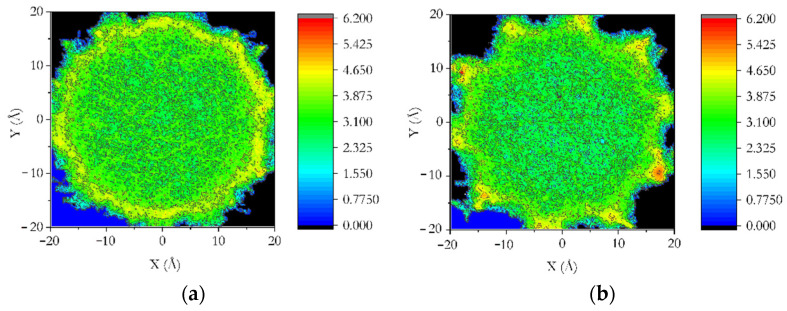
Distribution of 150 methane molecules over 75,000-time frames of MD simulations in (**a**) smooth and (**b**) undulated 4 nm cylindrical pore of amorphous silica.

Figure 4 summarizes the effects of pore size and the surface-to-volume ratio on the distribution of methane molecules within the pore. This distribution is plotted as a function of distance from the pore axis. A peak close to the pore surface can be seen representing an adsorbed layer, beyond which the distribution is without structure in most cases towards the pore center (smaller distances). Additionally, some methane molecules penetrate the pore surface and get trapped in the amorphous silica substrate. These molecules give rise to non-zero contributions in the distribution plots beyond the pore radii. The fraction of molecules in the adsorbed layer gets progressively more significant as the pore size decreases. This can have important implications for the dynamics of methane. Comparing the distribution in the undulated and smooth pores reveals the effect of the surface-to-volume ratio on the structure of adsorbed methane. The adsorption peak is broadened in the undulated pore, while its height is reduced compared to the peak in the smooth pore. The origin of this broadening of the pores could be the groves in the undulated pore surface that span a more extensive range of distances from the pore axis and can accommodate more adsorbed molecules. The undulated pore is also relatively more permeable to the adsorbed methane molecules, which gives rise to higher contributions beyond the pore radius than the smooth pore. Further insights can be obtained into the difference in the distribution of adsorbed methane molecules between the undulated and smooth pores by comparing the projection of adsorbed methane positions in the X-Y plane in the two cases, as shown in Figure 5.

Figure 5 compares the distribution of 150 molecules of methane in 75,000-time frames of the NVT simulation between the undulated and smooth pores. The plot in either panel represents the projection of a part of the simulation cell in the X-Y plane (perpendicular to the pore axis). The intensity (different colors) represents the logarithm of the number of molecules found at a given point in the X-Y plane + 1 (1 added to the number of molecules is a mathematical convenience to make the lower end of the scale equal to 0). More molecules are adsorbed on the pore surface (lighter yellowish hue) in the undulated pore compared to the smooth pore. Conversely, smaller molecules can be seen in the central pore region in the undulated pore. More methane molecules penetrated the pore surface in the undulated pore, evidenced by a more significant number of patches of color beyond the pore region. Another critical difference between the smooth and undulated pores is the structure of the adsorbed layer which forms a uniformly continuous circle in a smooth pore surface (see a constant line of light yellow hue in Figure 5a).

In contrast, in the undulated pore, this adsorbed layer is composed of somewhat isolated regions in the surface groves connected by a slightly low-intensity area. The adsorption within the groves on the surface of the undulated pore is relatively stronger than on the smooth pore surface. These strongly adsorbed methane molecules on the undulated pore surface give rise to a significant difference in the dynamical properties of methane compared to the smooth pores, as well as the properties probed by NMR reported above.

#### 3.4.2. Dynamics

**Figure 6:**
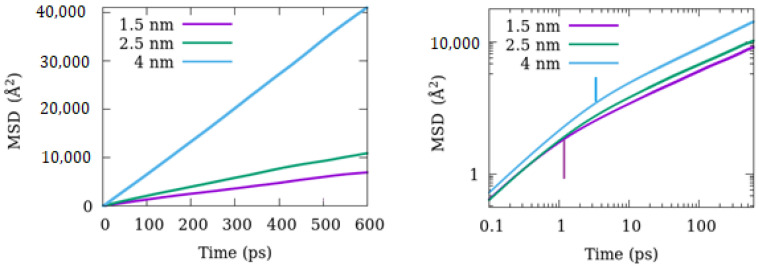
Mean squared displacement (MSD) of methane in cylindrical pores of amorphous silica at 346 K in a linear-linear (**left**) and log-log (**right**) plot. Vertical lines in the right plot mark the boundaries between the ballistic and diffusive regimes in the MSD curves of the corresponding color.

The dynamics of methane adsorbed in the amorphous silica pores were assessed by calculating the methane molecules’ mean squared displacement (MSD) from the trajectories obtained in the MD simulations. Figure 4 and Figure 5 demonstrate that some molecules penetrate the pore surface and get trapped within the amorphous silica substrate, becoming essentially immobile. Including them in the calculations of MSD can significantly underestimate the diffusion coefficient. Therefore, MSD was calculated by considering only those molecules that occupied the pore. Figure 6 shows the plots of MSD versus time for different pore sizes at 346 K in linear (left panel) and log-log (right panel) scales. The effect of pore size is readily apparent—the larger the pore, the larger the magnitude of displacement. The log-log (right panel) plot helps understand the nature of molecular motion. The MSD plot has roughly two regions, the first below ~1 ps that shows a faster increase with time and the second beyond this time where MSD varies with t slowly. The first region represents the ballistic motion that a molecule undergoes before colliding with another methane molecule or an atom of the substrate. The boundary separating the two regions, marked with vertical lines for the 1.5 nm and 4 nm pores with the corresponding color, shifts gradually to longer as we go from smaller to larger pores. This is because, as the pore gets more extensive, a typical molecule will have longer and longer distances available for the free motion before colliding with another atom or molecule. After encountering collisions with other molecules/atoms, the rate of increase of MSD with time slows down, and the motion becomes diffusive. This diffusive motion is seen in the second region beyond ~5 ps.

**Figure 7:**
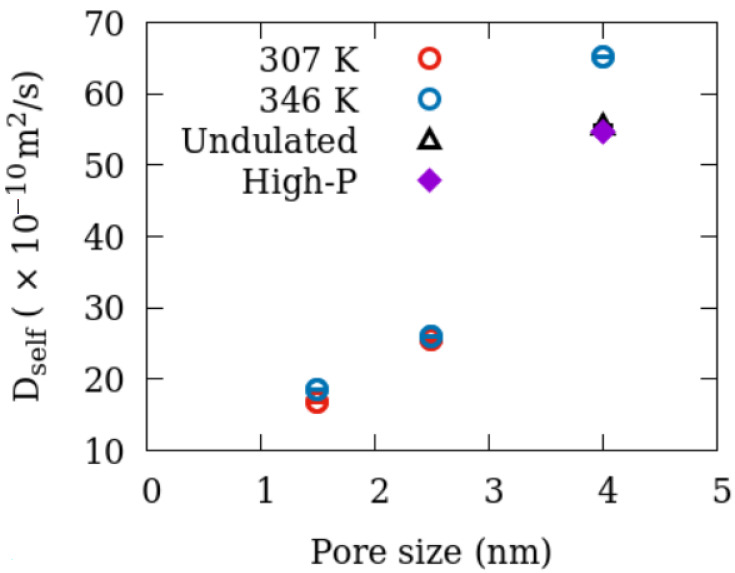
Self-diffusion coefficient of methane in the cylindrical pores of amorphous silica obtained from the simulations. All data in the 4 nm pore were obtained at 346 K.

Self-diffusion coefficient D_self_ can be obtained from the MSD vs. t plot as slope/(2d), where d is the number of dimensions in which the motion is allowed, and the slope is obtained from the data at extended times (typically hundreds of picoseconds). D_self_ was obtained this way for all the simulations listed in Table 1 and is plotted in Figure 7 as a function of pore size. Compared to all other factors investigated, the effect of temperature on D_self_ was observed to be minor in the 1.5 nm and 2.5 nm pores. Therefore, simulations in the 4 nm pore were carried out only at 346 K. As evident from the slope of MSD vs. time curves plotted in Figure 4, the pore size significantly affects the diffusion coefficient. Methane gets progressively faster in larger pores. In the 4 nm pore, the impact of increasing the surface-to-volume ratio and increasing the pressure 2-fold is similar. Both tend to suppress the motion of methane. While the effects of pressure could be due to crowding, a higher surface-to-volume ratio in the undulated pore is because of stronger adsorption resulting from a rougher surface. Using MD simulations, it has been found in other studies that the position-dependent diffusive motion of a layer adsorbed close to the pore wall in a strongly adsorbed fluid is slower [52]. In undulated pores, this adsorbed layer is thicker (see Figure 4b). The fraction of molecules in this layer being higher in the undulated pore, the overall diffusion coefficient in the higher surface-to-volume pore is smaller compared to a pore of the same size with smooth surfaces and hence a smaller surface-to-volume ratio.

## 4. Discussion

The change in the isotropic chemical shift positions of methane in engineered proxies is explained by changes in the microenvironment of methane molecules and structural distortion of methane by interaction with the surface of the pore walls. When the methane molecules are confined to the nanopores of the silica materials, at first, densification of the methane molecules happens on the pore walls, possibly via interaction with -OH groups. Hence, a layered structure would be formed by the methane molecules on the pore walls, as also found by MD simulations (Figure 4).

**Figure 8:**
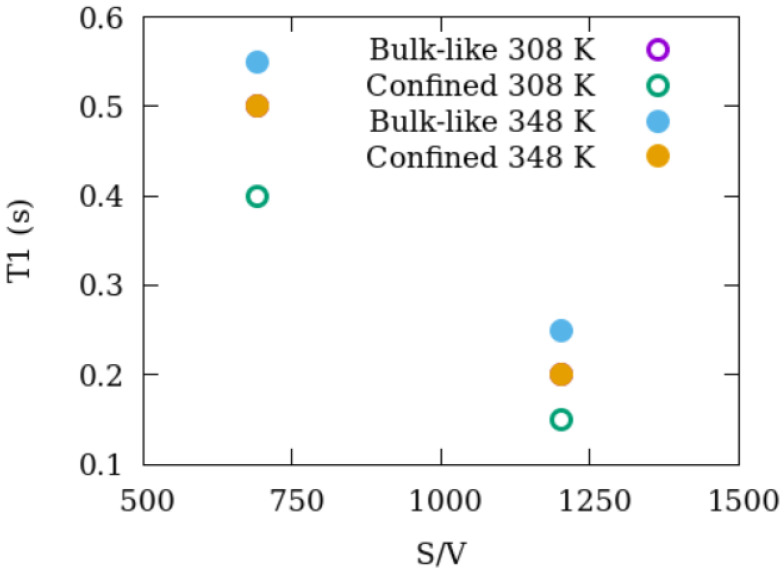
Change in the relaxation values of methane in mixtures with engineered nanoporous silica proxies. The open circles represent bulk-like methane, while the filled circles represent confined methane. 307 K for P = 28.2 and 112.7; 346 K for P = 32.6 and 130.3 bar.

The structural change is accompanied by changes in dynamics as revealed by experimental relaxation measurements and diffusion results derived from MD simulations. As seen in Figure 8 showing T_1_ as a suction of S/V, relaxation becomes faster with an increase in S/V. However, long-range diffusion gets slower because methane molecules get trapped in the groves of higher S/V material (see Figure 7). The methane molecules trapped in the groves of higher S/V material thus agitate with higher energy (smaller T_1_) but cannot go farther and exhibit a lower diffusion. Our combined study employing NMR experiments and MD simulations thus provides a complete picture of the dynamics (relaxation + diffusion) and structure of methane in silica pores.

## 5. Conclusions

In the presence of CO_2_ in mixtures of methane and nanoporous silica matrixes, when the amount of CO_2_ is significantly higher than that of methane, there might be “competition” between methane and CO_2_ molecules occupying the pore volume of the nanoporous silica materials. T_1_ measurements demonstrate that surface-to-volume ratios have a more significant effect on methane dynamics than pore diameter. We suggest that during the exposure of nanoporous silica materials, the cross-linked nanoporous silica starts swelling and enlarging the pore volume leading to an increase in dynamics revealed by longer T_1_ values. As tested and evidenced by exposure of engineered nanoporous silica proxies to only CO_2_, the swelling also alters the nanoporous silica materials’ original pore diameter and surface area. The potential explanation of the experimental results, such as the densification of methane on pore walls by interacting with -OH groups and the competition between CH_4_ and CO_2_ molecules concerning sorption to the pore walls, are further studied by MD simulations. For example, the changes in the intensity of NMR peaks and changes in isotropic chemical shift positions of methane are explained by either densification of CH_4_ and heterogeneity of the pore walls of porous silica engineered proxies and surface-to-volume (S/V) ratios of the porous silica systems. Nanoporous silica systems were built virtually for MD simulations to complement the experimental results. MD simulations provide insights into the structural distribution of methane molecules inside the cylindrical pores of amorphous silica. A strong, adsorbed layer of molecules is found close to the pore wall in all pores. In a 4 nm pore with a higher S/V ratio, this adsorbed layer is broader than that in the pore of the same diameter but with a lower S/V ratio. This more comprehensive adsorbed layer in the higher S/V ratio reduces diffusivity.

## Figures and Tables

**Table 1:** The number of methane molecules adsorbed in the amorphous silica pores at different temperature and pressure conditions. MD simulations were carried out on all the environmental conditions listed in the table.

Pore Size	Pore Surface	Pressure (bar)	Temperature (K)	Number of Methane Molecules
1.5 nm	Smooth	30	307	66
			346	66
2.5 nm	Smooth	30	307	88
			346	88
4.0 nm	Smooth	30	346	150
		60	346	305
	Undulated	30	346	150

**Table 2:** Sample conditions, compositions, and measured ^13^C NMR isotropic chemical shift values of methane in the pure state and mixtures with silica systems.

Sample	Pressure	Temperature (K)	Bulk (ppm)	Confined (ppm)	Amount of	Amount of Methane (mg)
	(bar)				Silica (mg)	
Pure methane	28.2	307	−10.45	-	-	0.0115
^13^C labeled	32.6	346	−10.45	-	-	0.0115
	112.7	307	−9.71	-	-	0.0452
	130.3	346	−9.71	-	-	0.0452
	>130.3	373	−9.90	-	-	0.0412
Methane + 1.5 nm silica	28.2	307	−10.71	−9.98	0.0274	0.0094
	32.6	346	−10.67	−10.14	0.0274	0.0094
	112.7	307	−9.86	−9.42	0.0274	0.0400
	130.3	346	−9.86	−9.50	0.0274	0.0400
	>130.3	373	−9.89	−9.54	0.0274	0.0400
Methane + 2.5 nm silica	28.2	307	−10.71	−9.91	0.0478	0.0080
	32.6	346	−10.71	−10.31	0.0478	0.0080
	56.4	307	−10.42	−9.92	0.0478	0.0219
	65.1	346	−10.42	−10.03	0.0478	0.0219
	112.7	307	−9.86	−9.50	0.0478	0.0398
	130.3	346	−9.86	−9.58	0.0478	0.0398
	>130.3	373	−9.86	−9.60	0.0478	0.0398

**Table 3:** Sample conditions, compositions, and measured ^13^C NMR isotropic chemical shift values of methane in mixtures with CO_2_ interacted with 2.5 nm silica with 0.0478 mg. Loading pressures of methane were 15 or 60 bar, while loading pressures of CO_2_ were 15, 45, and 60 bar.

Sample	Loading Pressure (bar)	Temperature (K)	Bulk (ppm)	Confined (ppm)	Amount of Methane + CO_2_ (mg)
	CH_4_ + CO_2_				0.0043 g methane + 0.0029 g CO_2_
methane	15 + 15	307	−10.71	−10.23	
methane	15 + 15	346	−10.70	−10.32	
CO_2_	15 + 15	307	125.38		
CO_2_	15 + 15	346	125.42		
					0.0043 g methane + 0.0487 g CO_2_
methane	15 + 45	307	−10.48	−10.13	
methane	15 + 45	346	−10.48	−10.18	
CO_2_	15 + 45	307	125.38		
CO_2_	15 + 45	346	125.42		
					0.0219 g methane + 0.0734 g CO_2_
methane	60 + 60	307	−9.83	−9.58	
methane	60 + 60	346	−9.85	−9.63	
CO_2_	60 + 60	307	125.42		
CO_2_	60 + 60	346	125.42		

**Table 4:** T_1_ relaxation values of methane for the bulk, bulk-like and confined states and in the presence of CO_2_.

		308 K	348 K
120 bar	pure CH_4_	0.35 s	0.55 s
2.5 nm Silica + methane 120 bar	bulk-like CH_4_	0.20 s	0.25 s
	confined CH_4_	0.15 s	0.20 s
1.5 nm Silica + methane 120 bar	bulk-like CH_4_	0.50 s	0.55 s
	confined CH_4_	0.40 s	0.50 s
2.5 nm Silica	bulk-like CH_4_	0.22 s	0.25 s
15 bar methane + 15 bar CO_2_	confined CH_4_	0.18 s	0.20 s
	CO_2_	0.05 s	0.35 s
2.5 nm silica	bulk-like CH_4_	0.95 s	1.05 s
15 bar methane + 45 bar CO_2_	confined CH_4_	0.85 s	1.00 s
	CO_2_	0.35 s	0.54 s
2.5 nm silica	bulk-like CH_4_	1.70 s	1.70 s
60 bar methane + 60 bar CO_2_	confined CH_4_	1.40 s	1.60 s
	CO_2_	0.85 s	0.90 s

**Table 5:** The surface area of 2.5 nm silica before and after exposure to CO_2_ or supercritical (sc) CO_2_.

Surface Area (m^2^/g)	2.5 nm Silica without Exposure to CO_2_	CO_2_ Exposed 2.5 nm Silica @ 30 bar, 35 °C, 2 h	sc CO_2_ Exposed 2.5 nm Silica @ 120 bar, 35 °C, 2 h
BET Surface area	1166.9	841.6	756.1
BJH Adsorption cumulative surface area	1097.6	734.1	651.7
BJH Desorption cumulative surface area	1263.4	841.5	719.6
Pore Volume (cm^3^/g)			
BJH Adsorption cumulative volume of pores	0.76	0.49	0.41
BJH Desorption cumulative volume of pores	0.88	0.55	0.47
Pore Size (Å)			
BJH Adsorption Average pore width	27.6	26.5	25.3
BJH Desorption Average pore width	27.8	26.3	26.2

## Data Availability

Not applicable.
